# A systematic review of the prevalence of burnout in orthopaedic surgeons

**DOI:** 10.1308/rcsann.2024.0009

**Published:** 2024-04-02

**Authors:** K Chahal, K Matwala

**Affiliations:** Mid and South Essex NHS Foundation Trust, UK

**Keywords:** burnout, professional, orthopaedic surgeons, systematic review, COVID-19

## Abstract

**Introduction:**

Professional burnout is a syndrome of emotional exhaustion, depersonalisation and low sense of personal achievement related to the workplace. Orthopaedic surgeons train and practise in highly demanding environments. Understanding up-to-date trends in burnout, particularly following the COVID-19 pandemic, is vital. For this reason, we carried out a systematic review on this topic.

**Methods:**

A scoping literature review of two databases was conducted. Two authors independently screened articles and conflicts were resolved by panel discussion. Articles pertaining to orthopaedic surgeons that used validated scales and were peer reviewed research were included. Non-English or abstract-only results were excluded.

**Results:**

A total of 664 papers were identified in the literature search and 34 were included in the qualitative review. Among 8,471 orthopaedic surgeons, the mean burnout prevalence was 48.9%. The wide range in rate of burnout between the studies (15–90.4%) reflected the variety in setting, subspecialty and surgeon grade. Common protective factors comprised dedicated mentorship, surgeon seniority, sufficient exercise and family support. Substance abuse, malpractice claims, financial stress and onerous on-call responsibilities were risk factors. Burnout prevalence during the COVID-19 pandemic was not noticeably different; there were a number of pandemic-associated risk and protective factors.

**Conclusions:**

Nearly one in two orthopaedic surgeons are burnt out. There is a paucity of data on the short and long-term impact of COVID-19 on burnout. Burnout has deep organisational, personal and clinical implications. Targeted organisational interventions are required to prevent burnout from irrevocably damaging the future of orthopaedic surgery.

## Introduction

Professional burnout is a syndrome of mental, physical and emotional exhaustion directly related to exposure to work-intensive professional environments over extended periods of time.^1^ A common consequence of burnout is feeling emotionally depleted, which results in depersonalisation and apathy towards one's job or colleagues. In surgery, it is clear that these consequences can be deleterious to patients, the multidisciplinary team and the surgeon themselves. Burnout is considered a syndrome comprising a triad of symptoms: loss of enthusiasm known as emotional exhaustion, diminished feelings of personal accomplishment and depersonalisation.^2^

Orthopaedic surgery is renowned worldwide for its emotional, physical and intellectual challenges. During and beyond training, litigation is ever present, with orthopaedic surgery being one of the most cited specialties in malpractice cases.^3^ Additionally, surgeons are experiencing greater bureaucratic demands while losing autonomy in their practice and having to deal with unfavourable media perception.^4^ Owing to these emotional stressors, it is inevitable that orthopaedic surgeons will suffer from burnout. This affects the individual surgeon, the healthcare system and the patient. Burnout has been shown to lead to more medical errors, unprofessional misdemeanours and reduced quality of life; for institutions, there is an increased turnover of staff and poor team morale, and patients ultimately receive substandard care.^5–8^

Developed in 1981, the Maslach Burnout Inventory (MBI) is a validated scoring system used to assess occupational burnout in physician and non-physician populations. With its 22-item Likert-scale questionnaire, it provides objective results for a somewhat subjective syndrome and is considered the gold standard for assessing burnout.^2^

In order to better address burnout in orthopaedic surgery, it is vital to assess the current literature. The aim of this study was therefore to provide an up-to-date scoping review of the current literature discussing burnout among orthopaedic surgeons.

## Methods

### Search strategy

A scoping review was conducted in line with the PRISMA (Preferred Reporting Items for Systematic reviews and Meta-Analyses) guidelines.^9^ One author (KC) searched the electronic databases of PubMed^®^ and Embase^®^ for publications up until 30 June 2023, employing the search term “(Orthopaedic OR Orthopedic) AND (Burnout)”. Reference lists of similar review articles were screened by KC and KM to identify any studies missed by our search. No date restrictions were imposed on the search but the search was limited to publications in the English language.

### Selection process

Articles were reviewed twice by both authors independently, initially by title/abstract screening and then by full-text review. The review process was facilitated by Covidence systematic review software (Veritas Health Innovation, Melbourne, Australia). Articles were included if they pertained solely to orthopaedic surgeons, used a validated scale to report a specific burnout rate in study participants and were peer-reviewed research. Articles were excluded if they were only available as an abstract or as a poster presentation, or if they were not in the English language. All discrepancies were resolved via panel discussion at the title/abstract and full-text review stages.

### Data collection

Study and author location, study date and cohort data were harvested. The primary outcome measure was reported rate of burnout. If not clarified in the paper, burnout was defined as an abnormal result in at least one of the emotional exhaustion or depersonalisation domains. Secondary outcome measures included causative factors for burnout, protective factors, and burnout rates during the COVID-19 pandemic.

## Results

**Figure 1:**
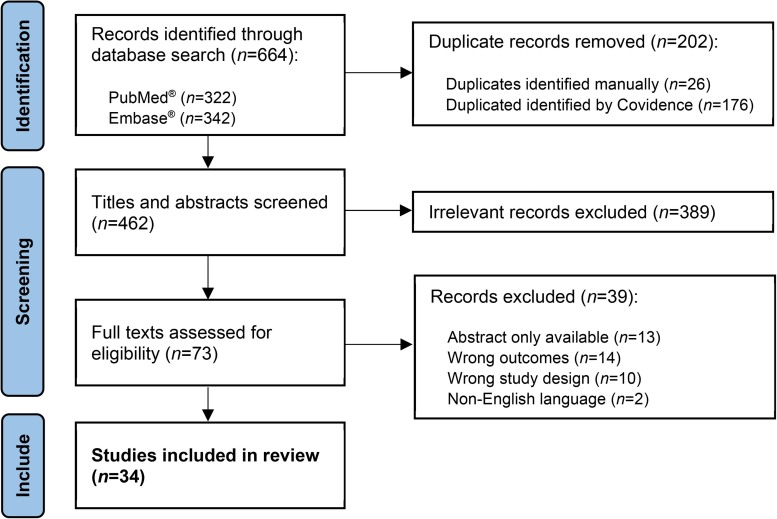
Flowchart of study selection

A total of 664 papers were identified in the literature search of the PubMed^®^ and Embase^®^ databases (Figure 1). Of these, 202 were removed owing to duplication and so 462 papers were initially screened. A total of 389 studies were subsequently deemed irrelevant by consensus or via agreement through panel discussion, leaving 73 to undergo full-text review. Thirty-nine studies were removed at this stage, most commonly because they did not report specific figures on burnout prevalence in their study population. This left 34 articles to be included in this review.^10–43^

### Rate of burnout

The overall burnout rates for the 34 studies are presented in Table 1. Data were collected between 2003 and 2023. The papers studied burnout rates among cohorts of orthopaedic surgeons from North America (*n*=13), Asia (*n*=9), Europe (*n*=8) or Oceania (*n*=4). This included multicentre and single-centre studies, and orthopaedic surgeons of all grades and subspecialties. Studies reported overall burnout rates using a number of validated questionnaires; overwhelmingly, the most utilised was the MBI (*n*=25). There was a wide range of reported burnout prevalence, ranging from 15% (in the US) to 90.4% (in the UK).

**Table 1:** Prevalence of burnout among orthopaedic surgeons

Study	Country	Data collection period	Validated scale(s) used	Number of surgeons	Seniority of surgeons	Burnout rate
Arora, 2014^10^	Australia	Nov – Dec 2012	MBI	51	Trainees	53%
Balch, 2011^11^	US	June 2008	MBI	500	All grades	45.6%
Bischoff, 2023^12^	France	June – July 2022	MBI	93	38 interns, 65 registrars	20%
Ebrahimpour, 2023^13^	Iran	May – June 2021	MBI	456	All grades	56.8%
Faivre, 2018^14^	France	Feb – April 2017	MBI	107	Residents	72.8%
Faivre, 2019^15^	France	Feb – April 2017	MBI	441	All grades	38.9%
Ghoraishian, 2022^16^	Iran	2019	MBI	180	Attending surgeons, fellowsand residents	50%
Ho, 2022^17^	Singapore	2022*	MBI	44	Residents	45.5%
Kollias, 2020^18^	Canada	Nov 2018	Expanded PWBI and PWBI	475	355 attending surgeons, 120 trainees	66.1%
Lesić, 2009^19^	Serbia	2009*	MBI	30	All grades	40%
Liang, 2021^20^	China	Aug – Sep 2019	MBI	700	All grades	73.7%
Lichstein, 2020^21^	US	2020*	Abbreviated MBI	661	Residents	52%
Martyn, 2022^22^	New Zealand	Aug 2021	MBI	116	Registrars	50.1%
Mir, 2021^23^	Australia, Canada, New Zealand, UK, US	March – May 2019	Mayo WBI and Stanford PFI	684	293 trainees,391 attending surgeons	58.6%
Morrell, 2020^24^	US	June – July 2018	Mini Z v2.0	595	All grades	49%
Nayar, 2024^25^	UK	Jan – March 2022	OBI	369	204 consultants, 100 registrars, 65 middle-grade surgeons	90.4%
Sadat-Ali, 2005^26^	Saudi Arabia	Sep 2003 – Oct 2004	MBI	69	All grades	59.4%
Saleh, 2007^27^	US	2007*	MBI	110	110 chairs or chiefs	36%
Sánchez-Madrid, 2005^28^	Spain	2005*	MBI	149	Attending surgeons	64.6%
Sargent, 2009^29^	US	2009*	MBI	648	384 residents,264 faculty members	44.8%
Shetty, 2017^30^	India	De 2013	MBI	299	Residents and more senior surgeons	23.1%
Siddiqui, 2018^31^	Pakistan	April – May 2018	APWA inventory	100	Trainees and consultants	80%
Simons, 2016^32^	US	2016*	MBI	38	27 residents, 11 staff surgeons	37%
Sochacki, 2019^33^	US	2019*	MBI	21	Residents and attending surgeons	61.9%
Somerson, 2020^34^	US	2018	MBI	203	Residents	38%
Thompson, 2023^35^	US	2023*	Abbreviated MBI	234	All grades	15%
van Vendeloo, 2014^36^	Netherlands	Nov 2011	MBI	105	Trainees	16.2%
Verret, 2021^37^	US	2021*	MBI	148	87 attending surgeons, 18 fellows, 43 residents	16%
Yu, 2020^38^	China	June 2017 – Oct 2018	MBI	643	Trainees	47%
Zheng, 2018^39^	China	2018*	MBI	202	All grades	85.1%
Al-Humadi, 2021^40^	US	April – May 2020	Abbreviated MBI	26	Residents/fellows andattending surgeons	26.9%
Barreto, 2021^41^	Brazil	Sep 2019 – June 2020	MBI	19	Residents	89.5%
Daryanto, 2022^42^	Indonesia	Aug 2020	MBI	51	Residents	58.8%
Lazarides, 2021^43^	US	April 2020	Stanford PFI	63	Attending surgeons and trainees	15.9%

*Where data collection period was not stated, year of publication is given.

APWA = American Public Welfare Association; MBI = Maslach Burnout Inventory; OBI = Oldenburg Burnout Inventory; PFI = Professional Fulfilment Index; PWBI = Physician Wellbeing Index; WBI = Wellbeing Index.

The highest rate of burnout (90.4%) was reported in the UK's only study, conducted in 2022 by Nayar *et al*, who described a cohort of 369 orthopaedic surgeons who answered a nationwide online survey; 68.3% of these had moderate burnout and 22.1% had high levels of burnout.^25^ They used the Oldenburg Burnout Inventory.

Thompson *et al* published their study most recently.^35^ Both emotional exhaustion and depersonalisation were reported at 21%, resulting in an overall burnout rate of 15% (the lowest rate reported in our review). In their study, burnout was most common among those who were employed solely by a hospital (i.e. not private or academic practice) (22%), those who had a practice duration of 5–10 years (17%) and those who specialised in oncology (25%). However, none of these characteristics had a statistically significant association with burnout.

Mir *et al* distributed the only multinational survey.^23^ They had the second largest cohort. In this study, relative risk for burnout was lowest in the US (0.93) and highest in Canada (1.25). New Zealand and UK surgeons had relative risks of 1.22 and 1.04 respectively.

### Risk and protective factors for burnout

Table 2 summarises the common risk and protective factors for burnout discussed in the papers. The factors can be split into three broad categories: personal, home life and workplace. The relationship between sex and burnout was inconclusive, as was that between ethnic background and burnout. Absence of protective factors (for example, not exercising or being more junior) in all cases conferred an increased burnout risk.

**Table 2:** Protective and risk factors for burnout

Factors decreasing burnout rate (protective)	Factors increasing burnout rate (risk)
• Having a dedicated mentor• Being able to pursue hobbies• Seniority	• Concerns about work–life balance due to long working hoursand onerous on-call responsibilities
• Confidential access to mental health professionals• Sufficient exercise (>4 hours/week)• Carrying out work in the private sector	• Excess alcohol/substance use• Financial worries most commonly due to excess debt but alsobecause of insufficient wages and incentives (e.g. poor pension)
• Psychological grit	• Undergoing malpractice claims
• Sufficiently staffed departments	• Inefficient electronic medical systems
• Supportive allied health professionals • Parent(s) being physicians	• Lack of feedback and displays of gratitude from seniors and patients
• Spousal support	• Regret relating to career choice

Each surgeon's mental health and experience of burnout is highly personal. Some studies focused on the impact of physical exercise and health optimisation, which were shown to decrease burnout.^16,17,25,35,37^ Thompson *et al* found that only 31% of surgeons were meeting the Centers for Disease Control and Prevention physical exercise guidelines; these individuals were less likely to be burnt out.^35^ Additionally, a consistent theme was that alcohol and substance abuse was associated with higher rates of depersonalisation burnout.^29,37,44^ Having the time to actively pursue hobbies and engaging with mental health support decreased emotional exhaustion burnout while financial worries and regret in career choice drove increased emotional exhaustion.^12,15,16,23,29,35^

Surgeons with strong family units were less likely to experience burnout.^11,17,25,28,29,35,38^ In a 2009 study of 648 participants, Sargent *et al* noted an association between having physician parents and lower rates of burnout.^29^ Engaging in a relationship where partners were able to make time to spend together and supported each other is highly protective.^11,14,21,29,38,39^ This can be limited by lengthy and irregular working hours, with on-call responsibility being a recognised risk factor for burnout.^11,12,17,20,21,24,25,32,34,37,39^

In professional burnout, the circumstances of one's work are a key factor in determining burnout risk. Many surgeons cite lack of feedback from seniors and concerns about performance as a source of distress.^14,17,21,22,25,29^ Conversely, trainees who experienced dedicated high-quality mentorship are less burnt out, as demonstrated by van Vendeloo *et al* in a cohort of 105 Dutch trainees on a modern educational programme^36^ and echoed by Sargent *et al.*^29^ Ghoraishian *et al* found that surgeons who worked solely in the public sector were six times more likely to develop burnout than those working in both the public and private sectors.^16^ Together with others, they also observed higher rates of burnout in junior surgeons than in their senior counterparts.^11,12,16,18,20,23,32,36,38^

### COVID-19 studies

Several studies carried out data collection during the COVID-19 pandemic and made specific reference to this period in their reports.^40–43^ Data from these studies can be seen in Table 3.

**Table 3:** Prevalence of burnout in COVID-19 studies

Study	Country	Data collection period	Burnout before pandemic	Burnout during pandemic
Al-Humadi, 2021^40^	US	April – May 2020	–	7/26 (26.9%)
Barreto, 2021^41^	Brazil	Sep 2019 – June 2020	44/52 (84.6%)	17/19 (89.5%)
Daryanto, 2022^42^	Indonesia	Aug 2020	–	30/51 (58.8%)
Lazarides, 2021^43^	US	April 2020	–	10/63 (15.9%)

The burnout rate in the four studies that focused on the pandemic ranged from 15.9% to 89.5%. Two studies were conducted in the US,^40,43^ one was conducted in South America^41^ and one in Asia.^42^ The mean overall burnout rate was 47.8% (median: 42.9%).

Barreto *et al* showed that despite a demonstrable rise in working hours during the pandemic, there was no statistically significant increase in rates of burnout.^41^ Meanwhile, Lazarides *et al* saw a high prevalence of concern regarding the post-COVID-19 future but a low rate of burnout (15.9%).^43^ Al-Humadi *et al* compared rates of burnout between orthopaedic surgery and other medical specialties during the pandemic.^40^ Orthopaedic physicians had the lowest incidence of poor mental health, with 3.8% reporting suicidal ideation. The average rate of redeployment from usual duties was 74.4% for all specialties and 66.7% among orthopaedic surgeons. This was associated with a lower rate of burnout in orthopaedic surgery than in specialties such as anaesthesiology and paediatrics. Daryanto *et al* compared orthopaedic surgery against other surgical specialties at a tertiary teaching hospital.^42^ Orthopaedics had the second highest rate of burnout at 58.8% (general surgery: 66.7%, urology: 40.0%).

The factors influencing burnout remained largely the same as displayed in Table 2. However, the four studies did note potential influences on burnout that were directly related to the pandemic as shown in Table 4. Orthopaedic surgery is a specialty with a heavy elective component; anxiety around suspended elective work and reduced training opportunities were potential causes of burnout.^40,41,43^ The rapid advancement and implementation of virtual learning technologies allayed these worries, and protected trainees from burnout due to concerns regarding lost educational time.^41^ Two studies cited adequate supply of personal protective equipment as an important measure to allay healthcare staff’s fears of contracting COVID-19 and passing it on to those close to them.^42,43^

**Table 4:** Protective and risk factors for burnout in COVID-19 studies

Factors decreasing burnout rate (protective)	Factors increasing burnout rate (risk)
• Adequate provision of personal protective equipment	• Fear of transmitting COVID-19 to family members
• Increased community/public support	• Uncertainty about future
• Emphasis on physician’s sense of duty and desire to help	• Stress from accumulating caseload due to cancelled elective work causing a backlog and lost training
• Implementation of remote learning technologies such as virtual reality to minimise educational impact	• Decreased interaction with colleagues

## Discussion

This review has collated data on burnout from a wide variety of settings, surgeon grades, subspecialties and time periods, including the COVID-19 pandemic. Among the total sample of 8,471 orthopaedic surgeons included in this review, there was a mean burnout prevalence of 49.6%. Shanafelt *et al* have been releasing periodic groundbreaking large-scale reviews on burnout among US physicians.^45^ In 2020, 38.2% of 7,510 physicians reported burnout; this was down from 43.9% in 2017, 54.4% in 2014 and 45.5% in 2011. This is in keeping with the results of our review, which covers studies including and beyond this timeline. Our review also mirrors the recent improvements in burnout rate that many of the more recent papers have reported.^13,34,35,37^

High prevalence and severity of burnout has deep organisational, clinical and societal implications. It is known that burnout increases unprofessional behaviours and worsens patient outcomes.^5–8^ Burnt out physicians are twice as likely to be involved in patient safety concerns and receive low satisfaction ratings from patients.^46^ One study included in our analysis, by Zheng *et al*, showed that surgeons suffering from burnout are more likely to lose their temper while operating.^39^ This can lead to hostile relationships with co-workers,^47^ which is an independent contributing factor that further fuels burnout.^13,17,21,28,34,36,37^ At the severe end of the spectrum, surgeons can develop suicidal thoughts.^11,48,49^

Nearly all the reviewed studies carried out subgroup analysis attempting to find associations between hypothetical risk and protective factors and burnout prevalence. Previous systematic reviews have identified key factors to be: long hours with excessive workload, and frequent on-call and night shifts; poor workplace relationships; anxiety about clinical competence; and poor work–life balance.^50,51^ In our analysis, themes were broadly categorised into personal, home life and workplace factors. Faivre *et al* carried out a multivariate analysis in which an abnormal General Health Questionnaire (GHQ-12), recent medical errors and not having a spouse conferred a risk of burnout.^14^ Thompson *et al* advocated optimising physical and mental health through regular health checks, practising wellbeing and exercising.^35^ Undergoing a malpractice claim,^52^ alcohol usage^17,21,29,37,44^ and long hours with frequent on-call commitments affecting work–life balance^11,12,17,20,21,24,25,32,34,37,39^ are seemingly associated with burnout risk whereas engaging in private practice,^11,12,15,16,28,30^ spousal support^11,14,21,29,38,39^ and mentorship^21,22,36,53^ are protective.

It is important to note that some studies conflicted on possible risk factors. Some were small single-centre studies with bivariate analysis; not all carried out multivariate statistical investigation. Factors could be causative but also a result of burnout. Risk factor analysis should therefore be interpreted with caution.

All of the COVID-19 studies carried out multivariate statistical analysis relating to the impact of potential risk factors and demographic data on burnout prevalence.^40–43^ While certain factors such as isolation from colleagues and fear of spreading the virus drove anxiety, none of the studies reported these to be associated with burnout, nor did they demonstrate an unusual spike in burnout. We hypothesise that the sentiment of gratitude from the public and an increased sense of duty among clinicians drove job satisfaction, which protected against burnout. However, the long-term psychological effects of the COVID-19 pandemic on healthcare staff are yet to be identified. Further research and monitoring for burnout are required following the pandemic.

When compared with the general population, physicians have notably higher rates of burnout (roughly 40% compared with 30%).^45^ This underlines the importance of active management of the issue in the medical sphere. In the papers included in this review, there was a consensus that orthopaedic surgeons are at higher risk of burnout in the earlier phases of their careers. Mir *et al* observed trainee status being associated with a higher risk of burnout^23^ while Sargent *et al* reported 56% burnout in residents and 28% in faculty members.^29^ If left unmanaged, these future surgeons are likely to leave the medical workforce before retirement, compounding workforce crises.^54^ This presents a key intervention point at a time when surgeons are often engaged in structured educational programmes, and are willing to engage with teaching and adapt practices.

On review of the medical literature, there is no single gold standard for preventive measures and management strategies for professional burnout. However, driven by increased awareness, there has been an upward trend in research attempting to address this problem. In the US, the National Academy of Medicine has published recommendations for a systems-based approach to prevent clinician burnout.^55^ Several systematic reviews summarise other system-based approaches throughout the world.^56–58^ Organisational support must be in place to provide sufficient resources to allow surgeons to carry out their roles; these should facilitate easy, confidential access to services in times of crisis (e.g. acute and chronic mental health counselling or support through a malpractice claim) and create the means for surgeons to have their performance valued or critiqued in a productive manner such as mentorship programmes.

## Conclusions

Our review has demonstrated that nearly one in two orthopaedic surgeons are burnt out. They are at risk of emotional exhaustion and depersonalisation causing (or in some cases, being fuelled by) decreased job satisfaction. Targeted organisational interventions are required to prevent burnout from irrevocably damaging the future of orthopaedic surgery.
